# Management of a massive thoracoabdominal impalement: a case report

**DOI:** 10.1186/1757-7241-18-57

**Published:** 2010-10-26

**Authors:** Haider Abbas

**Affiliations:** 1 Department of Anaesthesiology, CSM Medical University, Lucknow, India

## 

Dear Sir,

With great interest, I read the case report of Management of a massive thoracoabdominal impalement(SJTREM,2009, 17:50 (7 October 2009)[1]. The topic is interesting but the position of patient decided by the authors could have been modified so that the airway management, anaesthesia and surgery could have been made more conventional, convenient, speedy and less cumbersome.

Trauma remains a leading cause of death across all age groups, some of the injuries are dynamic and it is crucial for the Anaesthetists to have upto date understanding of Injury patterns, mechanisms, and pathophysiology to facilitate optimal management of these patients[2] because in some cases of thoracic Impalement Injuries chances of survival[3] are high. Early deaths are secondary to hypoxemia, airway obstruction, hemorrhage, haemothorax, cardiac tamponade and aspiration.

In this published case report the impaled iron angle was projecting in the anterior-posterior direction and the patient and iron angle were supported at all times and the authors decided to intubate the patient in semi-reclining position supported all the time by helpers, anesthetist stood on the stool to gain additional height and even left thoraco-abdominal incision needed to be given instead of conventional midline or paramedian Incision.

Peroperative management is very challenging in such cases and the position of patient is very crucial for the safe conduct of such cases. One of the options available is to place the patient in lateral position[4]. Different authors have described the use of fibreoptic intubation is sitting position[5]. This technique has limited value in emergency situations and may require more time than conventional laryngoscopy.

Position of the patient can be modified in such cases for safe peroperative management of patients. Operation theatre tables are composed of different attachments so that various positions(trendelenberg, anti-trendelenberg, sitting, lateral) can be made for different procedures. I am of the view that in this case the patient could have been placed in the supine postion after transfer from the ward with some additional help from the theatre staff by using gap (Figure 1) in the theatre table attachments where the Impaled rod can be placed and peroperative management can be done in more conventional, convenient and speedy manner (Figure 2).

**Figure 1:**
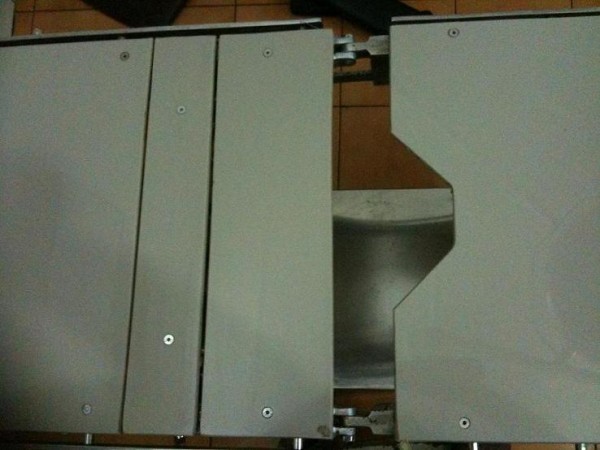
**Operation Theatre Table Top**. Still Image showing operation theatre table top with gap between the table attachments.

**Figure 2:**
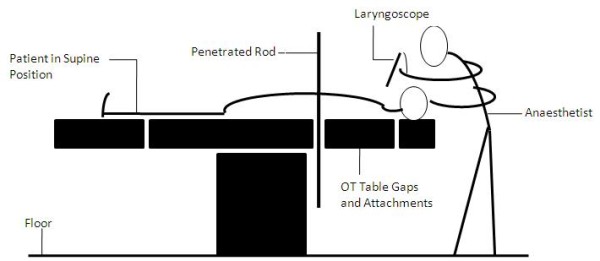
**Line diagram showing the patient and the anaesthetist's positions during Intubation**. The anaesthetist is standing on the floor while intubating the patient who is lying supine on the table with penetrated rod (passing through the thoraco-abdominal region) placed in the gap between the table attachments of the operation theatre table.

To summarize, the management of massive thoraco-abdominal impalement injuries can be made simpler by modifying the position of patient by making use of gaps in the theatre table attachments and placing the patient in conventional supine postion.

## Abbreviations

SJTREM: (Scandinavian Journal of Trauma, Resuscitation and Emergency Medicine)

## Competing interests

The author declares that they have no competing interests.
